# Parallel Implementation of Modeling of Fractional-Order State-Space Systems Using the Fixed-Step Euler Method

**DOI:** 10.3390/e21100931

**Published:** 2019-09-24

**Authors:** Rafał Stanisławski, Kamil Kozioł

**Affiliations:** Departament of Electrial, Control and Computer Engineering, Opole University of Technology, 45-758 Opole, Poland; kamilkoziol92@gmail.com

**Keywords:** fractional-order systems, Grünwald-Letnikov derivative, parallel computing

## Abstract

This paper presents new results in implementation of parallel computing in modeling of fractional-order state-space systems. The methods considered in the paper are based on the Euler fixed-step discretization scheme and the Grünwald-Letnikov definition of the fractional-order derivative. Two different parallelization approaches for modeling of fractional-order state-space systems are proposed, which are implemented both in Central Processing Unit (CPU)- and Graphical Processing Unit (GPU)-based hardware environments. Simulation examples show high efficiency of the introduced parallelization schemes. Execution times of the introduced methodology are significantly lower than for the classical, commonly used simulation environment.

## 1. Introduction

Fractional-order systems incorporating fractional-order derivatives (or differences) have attracted considerable research interest as their specific nature can be more adequate to describe some complex physical phenomena [1,2,3,4,5,6,7,8,9,10,11,12,13]. Since the fractional-order derivative is not defined at a point as in the case of its integer-order counterpart, impulse responses of fractional-order systems are not, in general, a class of exponential functions. In addition, a fractional-order derivative affects the properties of the system in the frequency domain. This is due to the fact that the modulus characteristic of a derivative of the fractional order α is increased by 20α dB per decade instead of 20 dB per decade for the integer-order derivative, whereas the phase characteristic is equal to πα/2. Therefore, the dynamic properties of the fractional-order system are more adjustable than for integer-order systems and can be more accurate in modeling various physical processes involving electrical circuits [4,6], thermal and diffusion processes [14,15], medicine [8,16], and others [17,18,19,20]. Among them, a lot of interest has been devoted to fractional-order generalizations of various entropy definitions and functions, i.e., Rényi entropy [11,21], Tsallis entropy [11,22], etc. The fractional generalizations of entropy definitions have led to different probability distribution functions as compared with the Shannon entropies [5]. However, the main problem encountered in the fractional-order systems is the fact that calculation of fractional-order derivative/difference may lead to computational problems. Namely, the Grünwald-Letnikov (GL) fractional-order derivative/difference, which is considered here, may lead to computational explosion related to the infinite summation. That is why truncated or finite-length implementation is used in approximators to the fractional-order derivative/difference [3,10]. Still, high modeling accuracy requirements often end up with high (upper) summation limits, which may be computationally burdensome for a single-core application. In spite of this, there is little discussion about using the multi-core architectures in calculations of fractional-order derivatives/differences. Therefore, the fractional-oriented parallel implementation issue is the main motivation for this paper.

Recently, trends in the development of more powerful computing hardware have focused on increased numbers of cores rather than the increased performance of an individual unit. For this reason, parallel computing has emerged as a research domain with the capability of meeting time requirements in both real-time applications and offline simulations. Despite the increased difficulty of coding, parallel programming has become very popular due to the ever-growing computational scale [23,24].

There are some papers considering the parallelization process for the fractional-order derivative/difference and fractional-order systems. In [25], a parallel computing application has been proposed for calculation of the Caputo derivative by use of the Adams–Bashforth–Moulton method. That method is extended to modeling of the fractional-order state-space system in [26]. Another approach for solving the Caputo derivative problem in the specific fractional-diffusion equation has been presented in [27], with a parallel algorithm based on linear tridiagonal equations. Yet another example is the use of parallel computing for solving nonlinear time-space fractional partial differential equations [28]. In that case, the authors have studied the efficiency of parallelizations for shared and distributed memory systems.

In this work, we use two strategies for parallel calculation of fractional-order state-space systems, the Grünwald-Letnikov derivative and the fixed-step discretization scheme. The first one is the use of a Central Processing Unit (CPU) with OpenMP (OMP) Application Programming Interface (API) [29]. The second one incorporates a Graphical Processing Unit (GPU) with Compute Unified Device Architecture (CUDA) API.

OMP is a set of compiler directives, library routines, and environment variables enabling shared-memory parallelism. With minimum latency, each thread or process can have direct access to memory throughout the system. It can be used to develop applications in programming languages C, C++, and Fortran on many platforms including Solaris, AIX, HP-UX, Linux, macOS, and Windows. OMP is supported by major computer hardware and software vendors, and is characterized by high-level parallelism, portability, scalability, and simplicity of use.

CUDA is an API developed by Nvidia Corporation. The CUDA platform (Nvidia Corporation, Santa Clara, CA, USA) is a software layer that provides a dramatic increase in computing performance by using the power of CUDA-enabled GPUs. It is designed to work with programming languages such as C, C++, and Fortran. In contrast to APIs like Direct3D and OpenGL, which require advanced skills in graphics programming, CUDA makes it easier for specialists to use GPU resources by use of virtual instruction set and parallel computational elements. It also supports programming frameworks such as OpenACC and OpenCL.

The paper is organized as follows. Having introduced the problem in Section 1, representations of the fractional-order derivative/difference and the fractional-order state-space system are presented in Section 2. Section 3 gives an introduction to the different parallel architectures that are considered in this paper. The detailed description, pseudo-code, and implementation method for the used parallel algorithms are also presented in Section 3. Simulation examples of Section 4 provide a comparative analysis of the introduced algorithm approaches as well as presented architectures. The analysis is accomplished in terms of high modeling accuracy and high time efficiency. Conclusions in Section 5 complete the paper.

## 2. Preliminaries

Consider a continuous-time linear time-invariant (LTI) state-space fractional-order system described by the following equations:

(1) $$\begin{array}{cc} D^{\alpha }x\left(t\right)= & Ax(t)+Bu(t),\\ y(t)= & Cx(t)+Du(t), \end{array}$$

where $A\in {ℜ}^{n\times n}$, $B\in {ℜ}^{n\times n_{u}}$, $C\in {ℜ}^{n_{y}\times n}$, and $D\in {ℜ}^{n_{y}\times n_{u}}$ are the system matrices; $n_{u}$ and $n_{y}$ are the number of inputs and outputs, respectively; *t* is the continuous time; and $D^{\alpha }=\mathrm{diag}\left\{D^{\alpha_{1}},\ldots ,D^{\alpha_{n}}\right\}$ is the matrix $D^{\alpha }\in {ℜ}^{n\times n}$ consisting of a fractional-order derivative $D^{\alpha_{i}}$ of order $\alpha_{i}$, i=1,…,n. The system (1) is commonly considered in a simplified commensurate fractional-order form, where the fractional-order $\alpha =\alpha_{i}$, i=1,2,…,n. In this case, $D^{\alpha }\in ℜ$ denotes a fractional-order derivative of order α. The fractional-order state-space system (1) is one of the most popular methods used to describe fractional-order processes. By using Equation (1), we can present fractional-order differential equations in a simple, matrix/vector form. Therefore, we can find many uses of the state-space system (1) in practical applications, e.g., in electrical circuits [4,6], in modeling of thermal and diffusion processes [12,13,15], in medicine [8,16], etc.

The fractional-order derivative is often described by using one of three definitions, that is, the Riemman-Liouville (RL), Caputo, or Grünwald-Letnikov (GL) definitions. Regarding the practical implementation point of view, in this paper, we use the Grünwald-Letnikov derivative defined as

(2) $$\begin{array}{ccc} D^{\alpha }{x\left(t\right)|}_{t=kh} & = & \lim_{h\to 0}\frac{1}{h^{\alpha }}\sum_{j=0}^{\infty }{\left(-1\right)}^{j}\frac{\Gamma (\alpha +1)}{j!\;\Gamma (\alpha -n+1)}x\left(t-jh\right), \end{array}$$

(3) =limh→01hα∑j=0∞(−1)jαjx(t−jh),

where α∈(0,2), *h* is the sampling period, Γ(.) denotes the Gamma function and αj, j=1,2,…, are the Newton binomial coefficients. Taking into account poor numerical feasibility of the Gamma function, we use the definition as in Equation (3). The GL definition is equivalent to the RL definition discretized by the use of the fixed-step discretization scheme. Additionally, in the case of homogenous initial value, the GL is equivalent to the Caputo definition. Moreover, by using specific correction coefficients, we can easily calculate the Caputo derivative by the use of the extended GL definition [30]. Therefore, the GL derivative based on finite length implementation can be used as the approximation of the Riemann–Liouville and Caputo derivatives. The main advantages of the GL definition are (1) they can be easily calculated in the recursive way, by using robust and numerically stable algorithms; and (2) an error of the finite-length GL approximation can be easily calculated, both for fractional-order difference/derivative and for the whole fractional-order system [31]. The main disadvantage of the finite-length GL is a low convergence rate. A detailed analysis of the effectiveness of finite-length GL as compared to other approximation methods can be found in [32]. In the literature, we can find several other definitions of fractional-order derivatives/differences [15,18,32]. In particular, He’s derivative is shown to provide a good numerical performance in specific applications [12,15]. Analysis of the state-space system using this definition will be a topic of our further work.

In order to solve the system (1) incorporating the GL derivative (3), a typical way is to use the simple fixed-step Euler method for calculation of fractional-order equation. Assuming that x(l)=0∀l≤0, we obtain

(4) Dαx(t)|t=kh≈1hα∑j=0k(−1)jαjx(t−jh),

or using a discrete-time formulation for k=0,1,…

(5) $$D^{\alpha }{x\left(t\right)|}_{t=kh}\approx \frac{\Delta^{\alpha }x_{k}}{h^{\alpha }},$$

where $x_{k}$ is the state vector defined in discrete time *k* and $\Delta^{\alpha }x_{k}$ denotes the discrete-time fractional difference

(6) $$\Delta^{\alpha }x_{k}=\sum_{j=0}^{k}P_{j}\left(\alpha \right)x_{k}q^{-j}=x_{k}+\sum_{j=1}^{k}P_{j}\left(\alpha \right)x_{k}q^{-j}\;k=0,1,\ldots$$

with α∈ (0,2), $q^{-1}$ being the backward shift operator and

(7) Pj(α)=(−1)jαj.

Note that the Newton binomials can be calculated by use of the simple, well-known formula

(8) αj=1j=0α(α−1)...(α−j+1)j!j>0.

Usually, in the fractional-order state-space systems, we use a forward-shifted form of the fractional-order difference (see e.g., [7]).

(9) $$\Delta^{\alpha }x_{k+1}=Ah^{\alpha }x_{k}+Bh^{\alpha }u_{k}.$$

(10) $$y_{k}=Cx_{k}+Du_{k}.$$

Combining Equations (6) and (9), we obtain the formula for calculating a fractional-order discrete-time state equation:

(11) $$x_{k+1}=\left(Ah^{\alpha }+\alpha I\right)x_{k}-\sum_{j=2}^{k+1}P_{j}\left(\alpha \right)x_{k-j+1}+Bh^{\alpha }u_{k}.$$

Note that each incoming sample of the signal $x_{k}$ increases the complexity of both the fractional-order difference (Equation (11)) and the whole fractional-order system (Equations (9) and (10)). This leads to the computational explosion for k→∞. To avoid this, a finite-length version of the fractional-order difference is used (see e.g., [7,10,33])

(12) $$\Delta_{L}^{\alpha }x_{k}=x_{k}+\sum_{j=1}^{L}P_{j}\left(\alpha \right)x_{k-j}\;k=0,1,\ldots ,$$

where *L* is the upper bound for *j*.

Now, combining Equations (9) and (12), we obtain the finite-length formula for calculation of the state equation

(13) $$x_{k+1}=\left(Ah^{\alpha }+\alpha I\right)x_{k}-\sum_{j=2}^{L}P_{j}\left(\alpha \right)x_{k-j+1}+Bh^{\alpha }u_{k}.$$

It is important to note that the results presented in Equations (12) and (13) for k>L define the approximations of the fractional-order difference and fractional-order state-equation, respectively.

*Problem formulation:* It is important that convergence of the series $P_{j}\left(\alpha \right)$ depends on the order α and is quite slow, in particular for a low value of α. Therefore, accurate approximation of Equation (5), and consequently Equation (12), requires a very high implementation length *L*. Exemplary norm $H\left(\alpha ,L\right)=\left|\right|\Delta_{L}^{\alpha }1\left(t\right)-\Delta^{\alpha }1\left(t\right){\left|\right|}_{L_{\infty }}$, where 1(t) is the Heaviside step function, is H(0.5,3180)=0.01, but to obtain the similar accuracy for α=0.3 we need as high a length as *L* = 2,000,000 (i.e., *h*(0.3, 2,000,000) = 0.01). Moreover, implementation of the fractional-order difference into the state-space system may require much higher *L* to obtain the same accuracy [31]. Therefore, real-time applications of both fractional-order difference and fractional-order state-space system for ’fast’ systems (with small sampling periods *h*) may require realization of the parallelization scheme for the calculation process.

## 3. Parallel Algorithms

Firstly, we introduce a parallel method for calculating the fractional-order difference. In the next step, the method is extended to the calculation of the fractional-order state-space system. Finally, a new hierarchical parallelization scheme for the calculation of the fractional-order system is proposed. It is important to note that the fractional-order difference incorporated into the fractional-order system leads to computational complication of the state equation only. The calculation of this equation, as is presented in the previous section, constitutes a computational burden process. In contrast, the output equation of the fractional-order system is the same as for the integer-order case and is based on simple vector/matrix operations. Therefore, we consider the calculation of the state equation only.

### 3.1. Fractional-Order Difference

**Figure 1:**
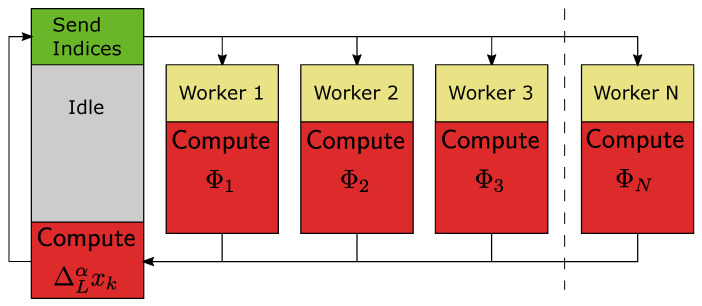
Block diagram of the calculation process.

Consider the fractional-order difference of Equation (12). In order to implement the parallelization scheme, we have to divide the summation process into *N* independent parts

(14) $$\begin{array}{ccc} \Delta_{L}^{\alpha }x_{k} & = & x_{k}+\sum_{i=1}^{N}\left[\sum_{j=\lfloor (i-1)L/N\rfloor +1}^{\left\lfloor iL/N\right\rfloor }P_{j}\left(\alpha \right)x_{k-j}\right], \end{array}$$

(15) $$\begin{array}{cc} = & x_{k}+\sum_{i=1}^{N}\Phi_{i}, \end{array}$$

where *N* is the number of parts/workers, ⌊.⌋ denotes the floor function, and

(16) $$\Phi_{i}=\sum_{j=\lfloor (i-1)L/N\rfloor +1}^{\left\lfloor iL/N\right\rfloor }P_{j}\left(\alpha \right)x_{k-j}=P_{i}X_{i}^{T}$$

with $P_{i}=\left[P_{\lfloor (i-1)L/N\rfloor +1}\left(\alpha \right),\;\ldots ,\;P_{\left\lfloor iL/N\right\rfloor }\left(\alpha \right)\right]$, and $X_{i}^{T}=\left[x_{k-\lfloor (i-1)L/N\rfloor -1},\;\ldots ,\;x_{k-\lfloor iL/N\rfloor }\right]$, i=1,…,N. Now, in the parallelization process we delegate calculation of elements $\Phi_{i}$ on particular workers. Finally, a block diagram of the calculation process for the fractional-order difference is presented in Figure 1 and the calculation algorithm is presented as Algorithm 1.

**Algorithm 1:** Parallel algorithm for one-step computation of fractional-order difference.

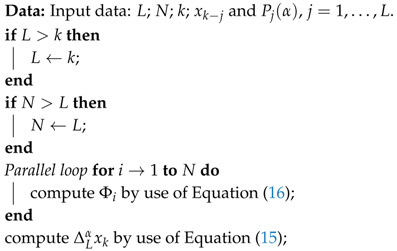

**Remark** **1.** *Note that Equation (12) can be considered as both a) finite implementation of the fractional-order difference when L<t and b) a fractional-order difference when L=t. Consequently, Algorithm 1 represents a parallel implementation for both cases.*

**Remark** **2.** *The number of workers N used in Algorithm 1 is bounded by the following condition N≤min(L,k). In addition, from a feasibility point of view, the number of workers N should be less than physical cores in the hardware environment.*

The calculation process of Figure 1 is a master/slave approach, with a master process (Worker 0) and *N* slave processes (Workers 1,…,N). A similar approach for the Caputo-based fractional-order state-space system is presented in [26].

### 3.2. Fractional-Order System

The parallelization algorithm presented in the previous subsection can be immediately applied to the fractional-order state-space system. In this case, one-step calculation process is as follows:

(17) $$\begin{array}{ccc} x_{k+1} & = & \left(Ah^{\alpha }+\alpha I\right)x_{k}+Bh^{\alpha }u_{k}-\sum_{i=1}^{N}\left[\sum_{j=\lfloor (i-1)(L-1)/N\rfloor +1}^{\left\lfloor i(L-1)/N\right\rfloor }P_{j+1}\left(\alpha \right)x_{k-j}\right], \end{array}$$

(18) $$\begin{array}{cc} = & \left(Ah^{\alpha }+\alpha I\right)x_{k}+Bh^{\alpha }u_{k}-\sum_{i=1}^{N}\Phi_{i}, \end{array}$$

where

(19) $$\Phi_{i}=\sum_{j=\lfloor (i-1)(L-1)/N\rfloor +1}^{\left\lfloor i(L-1)/N\right\rfloor }P_{j+1}\left(\alpha \right)x_{k-j}=P_{i}X_{i}^{T}$$

with $P_{i}=\left[P_{\lfloor (i-1)(L-1)/N\rfloor +2}\left(\alpha \right),\;\ldots ,\;P_{\lfloor i(L-1)/N\rfloor +1}\left(\alpha \right)\right]$, and $X_{i}^{T}=\left[x_{k-\lfloor (i-1)(L-1)/N\rfloor },\;\ldots ,\;x_{k-\lfloor i(L-1)/N\rfloor }\right]$, where $P_{i}\in {ℜ}^{1\times \lfloor i(L-1)/N\rfloor -\lfloor (i-1)(L-1)/N\rfloor }$ and $X_{i}\in {ℜ}^{\lfloor i(L-1)/N\rfloor -\lfloor (i-1)(L-1)/N\rfloor \times n}$. Note that each row of the vector $\Phi_{i}$ is quite similar to those of Equation (16), the difference being only the single forward time shift. Note that, in this case, particular workers calculate $\Phi_{i}$, i=1,…,N, and the master worker calculates the next step of system states (Equation (18)) and the output signal on the basis of Equation (1). The parallelization process for the above scheme is presented in Algorithm 2. As in Algorithm 1, the number of workers *N* used in Algorithm 2 is bounded by the condition given in Remark 2.

**Algorithm 2:** Parallel algorithm for one-step calculation of fractional-order system.

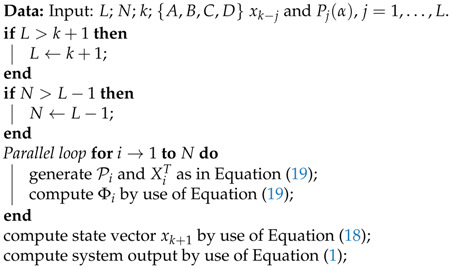



Furthermore, we can calculate the next values of state vector $x_{k+1}$ on the basis of the following equations:

(20) $$\begin{array}{ccc} x_{k+1} & = & \tilde{P}{\tilde{X}}_{k}, \end{array}$$

where

(21) $$\begin{array}{ccc} \tilde{P} & = & \left[A_{\alpha },\;{\tilde{P}}_{2}\left(\alpha \right),\;\ldots ,\;{\tilde{P}}_{L}\left(\alpha \right),\;Bh^{\alpha }\right], \end{array}$$

(22) $$\begin{array}{ccc} {\tilde{X}}_{k} & = & {\left[x_{k}^{T},\;x_{k-1}^{T},\;\ldots ,\;x_{k-L}^{T},\;u_{k}^{T}\right]}^{T} \end{array}$$

with $A_{\alpha }=A-I\alpha$, ${\tilde{P}}_{j}\left(\alpha \right)=IP_{j}\left(\alpha \right)$, j=2,…,L. The sizes of matrices are as follows: $\tilde{P}\in {ℜ}^{n\times nL+n_{u}}$ and ${\tilde{X}}_{k}\in {ℜ}^{1\times nL+n_{u}}$. In the case of modeling the noncommensurate-order system, we can still use Equation (20), where the elements ${\tilde{P}}_{j}\left(\alpha \right)$, j=2,…,L are substituted by ${\tilde{P}}_{j}=\mathrm{diag}\left\{P_{j}\left(\alpha_{1}\right),\;\ldots ,\;P_{j}\left(\alpha_{n}\right)\right\}$.

**Figure 2:**
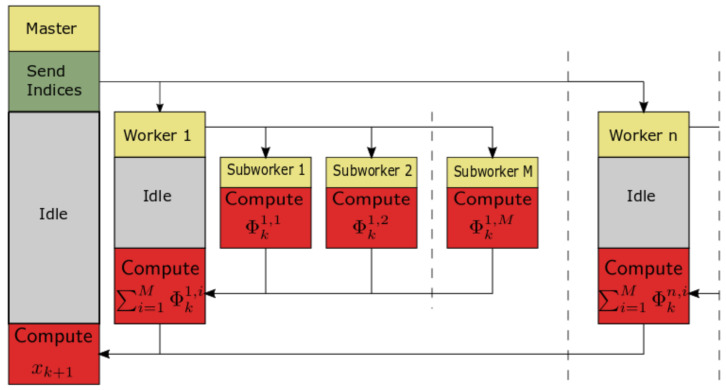
Block diagram of the hierarchical parallelization scheme.

Note that Equation (20) is a simple matrix form of the state equation, but the dimensions of the matrix $\tilde{P}$ and vector ${\tilde{X}}_{k}$ are large. Therefore, using Equation (20) is not effective from the computational complexity point of view. Taking into account that $\tilde{P}$ is the sparse matrix, we can present Equation (20) in a more computationally effective form as

(23) $$x_{k+1}=\left[\begin{array}{c} x_{k+1}^{1}\\ \vdots \\ x_{k+1}^{n} \end{array}\right]=\left[\begin{array}{c} \Phi_{k}^{1}\\ \vdots \\ \Phi_{k}^{n} \end{array}\right],$$

where $\Phi_{k}^{i}={\tilde{P}}^{i}{\tilde{X}}_{k}^{i}$ and

(24) $$\begin{array}{ccc} {\tilde{P}}^{i} & = & \left[a_{i,1}^{\alpha },\;...,\;a_{i,n}^{\alpha },\;P_{2}\left(\alpha \right),\;...,\;P_{L}\left(\alpha \right),\;b_{i,1},\ldots ,\;b_{i,n_{u}}\right], \end{array}$$

(25) $$\begin{array}{ccc} {\tilde{X}}_{k}^{i} & = & {\left[x_{k}^{T},\;x_{k-1}^{i},\;\ldots ,\;x_{k-L}^{i},\;u_{k}^{T}\right]}^{T} \end{array}$$

with i=1,…,n, $a_{i,j}^{\alpha }$, j=1,…,n are the entries in the *i*-th row of the matrix $A_{\alpha }$ and $b_{i,j}$ are the elements in *i*-th row of the matrix *B*, respectively. Now, we can apply the parallelization algorithm to Equation (23) and calculate $\Phi_{k}^{i}$ in various processes. Moreover, we can implement the parallelization scheme to calculate particular $\Phi_{k}^{i}$, i=1,…,n, as follows

(26) $$\Phi_{k}^{i}=\sum_{j=1}^{M}\Phi_{k}^{i,j},$$

where

(27) $$\Phi_{k}^{i,j}=\sum_{m=\lfloor \left(j-1\right)\frac{n+L+n_{u}}{M}\rfloor +1}^{\left\lfloor j\frac{n+L+n_{u}}{M}\right\rfloor }{\tilde{p}}^{i,m}{\tilde{x}}_{k}^{i,m}$$

with ${\tilde{p}}^{i,m}$ and ${\tilde{x}}_{k}^{i,m}$, $m=1,\ldots ,n+L+n_{u}$ being the *m*-th elements of the vectors ${\tilde{P}}^{i}$ and ${\tilde{X}}_{k}^{i}$, respectively. As a result of the parallelization scheme for Equations (23)–(27), we obtain a kind of a hierarchical parallelization process. Firstly, the calculation for time step *k* is divided into *n* parts, computing $\Phi_{k}^{i}$, i=1,…,n. Then, the calculation of each $\Phi_{k}^{i}$ is distributed on *M* subtasks, calculating $\Phi_{k}^{i,j}$, j=1,…,M. The block diagram of the hierarchical parallelization scheme is presented in Figure 2 and the pseudo-code is presented in Algorithm 3.

**Algorithm 3:** Hierarchical parallel algorithm for one-step calculation of fractional-order system.

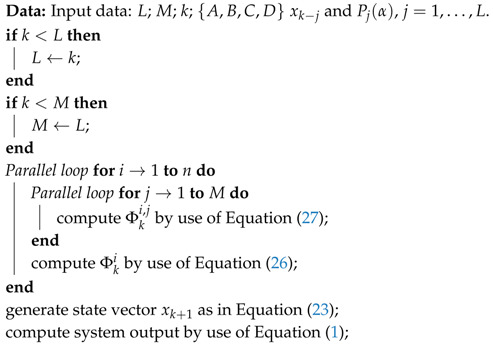



Finally, in the hierarchical parallelization algorithm presented above, to calculate state vector $x_{k+1}$, we use (M+1)×n+1 workers.

**Remark** **3.** *The number M used in Algorithm 3 has to fulfill the following condition: M≤min(L,k).*

The parallelization methods for the fractional-order difference in Equation (14) and for the fractional-order system in Equation (17) can be used for a wide spectrum of multi-core processors. On the other hand, the hierarchical parallelization method presented in Figure 2 requires a relatively high number of cores, therefore, the method is recommended in case of using Massively Parallel Processors (MPP).

## 4. Simulation Examples

In this section, we present the implementation results of parallelization methods both for the fractional-order difference and the fractional-order state-space system. For analysis, the CPU- and GPU-based hardware environments have been used.

In simulation experiments, we consider the fractional-order state-space system {A,B,C,D} as follows:

$$\begin{array}{cc} A= & \left[\begin{array}{cccccccc} -3.6557 & -8.5928 & -8.9663 & -5.2783 & -1.6870 & -0.2600 & -0.0171 & -0.0012\\ 1 & 0.8 & 0 & 0 & 0 & 0 & 0 & 0\\ 0 & 1 & 0.8 & 0 & 0 & 0 & 0 & 0\\ 0 & 0 & 1 & 0.8 & 0 & 0 & 0 & 0\\ 0 & 0 & 0 & 1 & 0.8 & 0 & 0 & 0\\ 0 & 0 & 0 & 0 & 1 & 0.8 & 0 & 0\\ 0 & 0 & 0 & 0 & 0 & 1 & 0.8 & 0\\ 0 & 0 & 0 & 0 & 0 & 0 & 1 & 0.8 \end{array}\right],\\ B= & {\left[\begin{array}{cccccccc} 0 & 0 & 0 & 1 & 0 & 1 & 0 & 0 \end{array}\right]}^{T},\;C=\left[\begin{array}{cccccccc} 0 & -1 & 0 & 0 & 1 & 0 & 0 & 0 \end{array}\right],\;D=\left[\begin{array}{c} 0 \end{array}\right],\\ \alpha = & \;0.7 \end{array}$$

with both the fractional-order difference and fractional-order system excited by the Heaviside step function u(t)=1(t).

### 4.1. CPU-Based Hardware

In case of calculation on the CPU-based hardware, numerical simulations are carried out on a computing node equipped with the Ubuntu 14.04 (Canonical Ltd., London, UK) operating system and an Intel Xeon E5−2650 v3 CPU (Intel Corporation, Santa Clara, CA, USA) with a basic frequency of 2.3 GHz. The hardware system offers 10 physical cores (20 threads based on the hyperthreading technology). During the simulations, we use one thread per physical core only, since the hyperthreading technology is not suitable in our task (see [34,35]). All the calculation algorithms in this subsection are implemented by use of the C++ programming language and the OMP library. The OMP library is based on the shared memory concept, therefore, explicit data distribution techniques are not desirable in this case. For evaluation of parallelization efficiency, solely the simulation times have been taken into consideration because all the methods provide the same calculation results.

**Figure 3:**
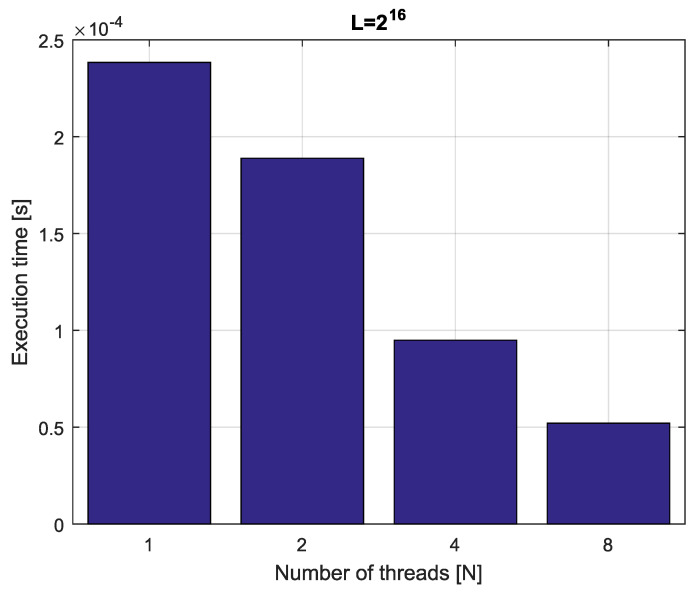
Execution times for fractional-order difference with implementation length $L=2^{16}$.

**Figure 4:**
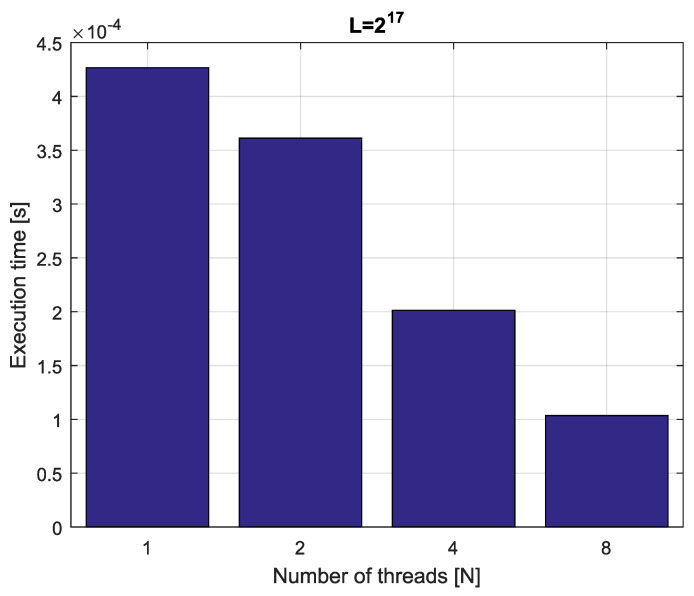
Execution times for fractional-order difference with implementation length $L=2^{17}$.

**Figure 5:**
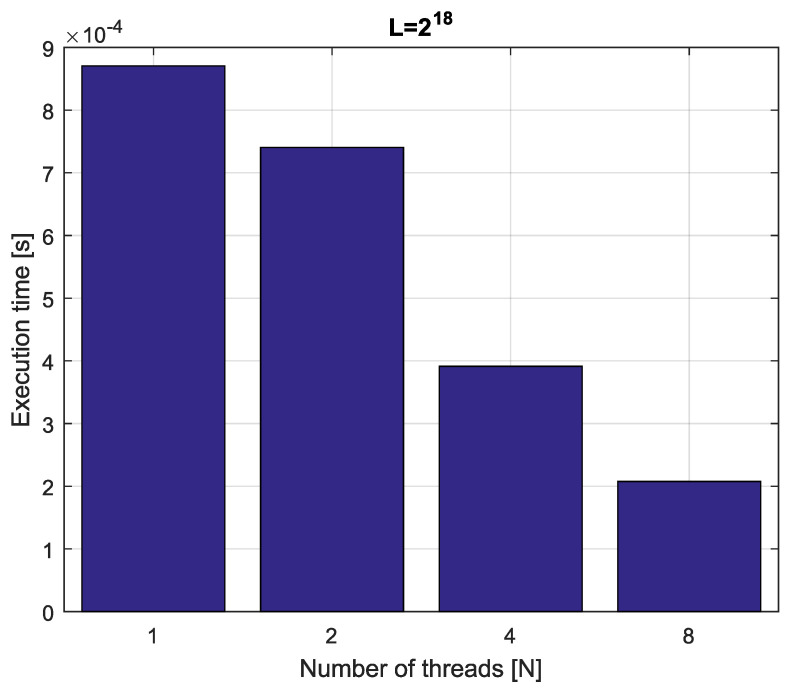
Execution times for fractional-order difference with implementation length $L=2^{18}$.

**Figure 6:**
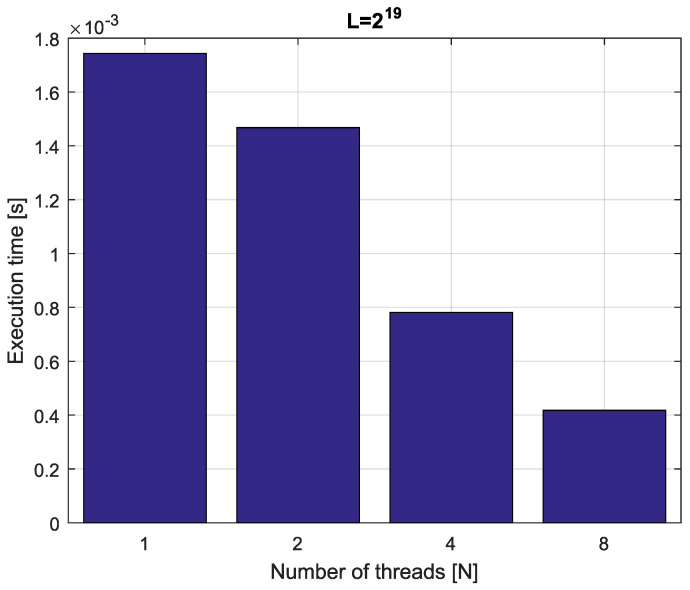
Execution times for fractional-order difference with implementation length $L=2^{19}$.

**Example** **4.** *Consider fractional-order difference (12) with α=0.7. The difference is implemented by use of the parallelization Algorithm 1. The simulation times for various implementation lengths $L=[2^{16},2^{17},2^{18},2^{19}]$ and various numbers of cores N=[1,2,4,8] are presented in Figure 3, Figure 4, Figure 5 and Figure 6.*

*As we can see in Figure 3, Figure 4, Figure 5 and Figure 6, the parallelization process decreases execution times of the one-step calculation process for fractional-order difference. The speedup for 8 cores varies from S=4.17 for $L=2^{19}$ to S=4.58 for $L=2^{16}$. So, the acceleration is similar for the considered lengths.*

**Figure 7:**
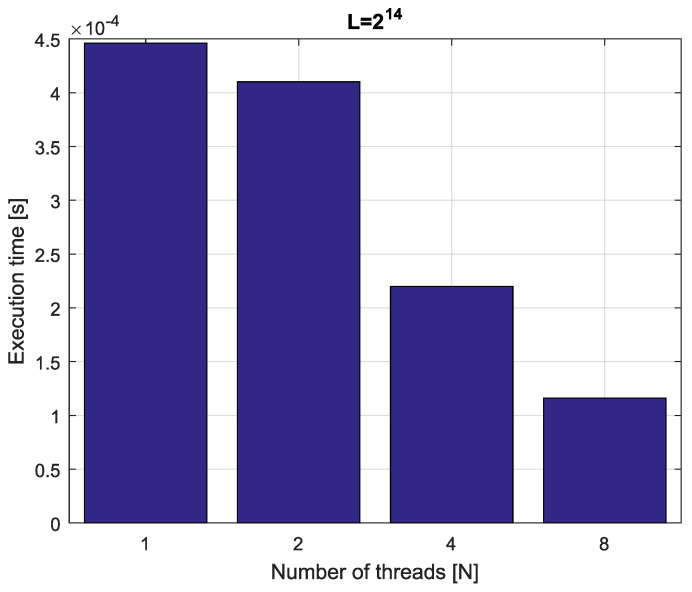
Execution times for fractional-order state-space system with implementation length $L=2^{14}$.

**Figure 8:**
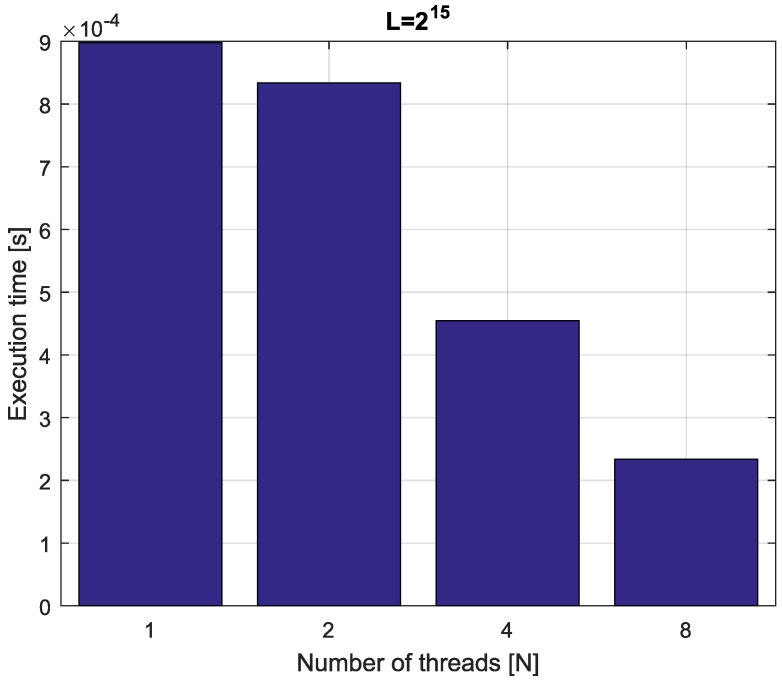
Execution times for fractional-order state-space system with implementation length $L=2^{15}$.

**Figure 9:**
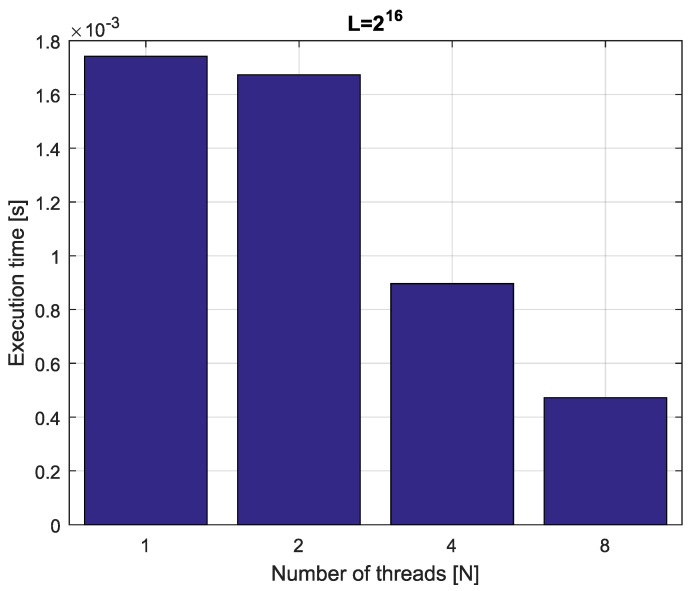
Execution times for fractional-order state-space system with implementation length $L=2^{16}$.

**Figure 10:**
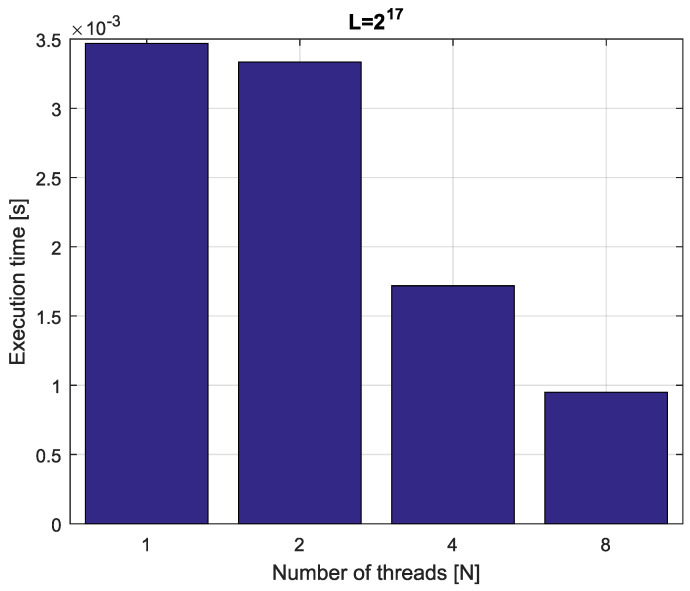
Execution times for fractional-order state-space system with implementation length $L=2^{17}$.

**Example** **5.** *Consider the fractional-order state-space system presented in the introduction of Section 4. The system is calculated by the parallel scheme introduced in Algorithm 2. The calculation process is executed in CPU-based hardware for implementation lengths $L=[2^{14},2^{15},2^{16},2^{17}]$. Results in terms of execution times for number of cores N=[1,2,4,8] are presented in Figure 7, Figure 8, Figure 9 and Figure 10.*

*We can see from Figure 7, Figure 8, Figure 9 and Figure 10 that, again, the parallelization process decreases execution times of the one-step calculation process for the fractional-order system. The speedup for 8 cores in this case varies from S=3.65 for $L=2^{17}$ to S=3.84 for $L=2^{14}$. This means, again, that the effectiveness of the parallelization is similar for the considered lengths.*

*We compared results obtained by the introduced methodology with time effectiveness of model implementation in the Matlab environment. In the case of using a single-core method, we obtain times from $1.0\times 10^{-3}$ for $L=2^{14}$ to $5.4\times 10^{-3}$ for $L=2^{18}$, therefore, the times are significantly higher than in the case where the OpenMP environment is used. Moreover, the implementation of parallel computing by the use of Parallel Computing Toolbox increases calculation times compared to a single-core approach. This is a result of the specific construction of CPU-based parallelization methods in the Matlab environment.*

### 4.2. GPU-Based Hardware

In the case of GPU-based implementation, we use hardware with two Tesla K80 (Nvidia Corporation, Santa Clara, CA, USA) accelerators with a dual-GPU design that consists of 4992 Nvidia CUDA threads, 24GB of GDDR5 memory, 480GB/s aggregate memory bandwidth, and up to 2.91 teraflops of double-precision operations. Accelerators operate on a computing node equipped with the operating system Windows Server 2012 R2 (Microsoft Corporation, Redmond, WA, USA) and an Intel Xeon E5−2683 v3 CPU (Intel Corporation, Santa Clara, CA, USA) with a basic frequency of 2.0 GHz. In contrast to OMP, CUDA does not support globally shared memory, so data distribution and memory allocation must be performed manually by proper data transfers between all processing units. CUDA enables the overlapping of some operations without losing much performance, but still, improper management of data distribution can result in poor time results. CUDA GPUs have many parallel processors grouped into Streaming Multiprocessors (SMs), creating a grid of threads arranged within blocks. Each SM can run multiple concurrent thread blocks. Tesla K80 GPU can support up to 1024 active threads in one working block. To take full advantage of all these threads, the program code must be executed with multiple thread blocks.

**Figure 11:**
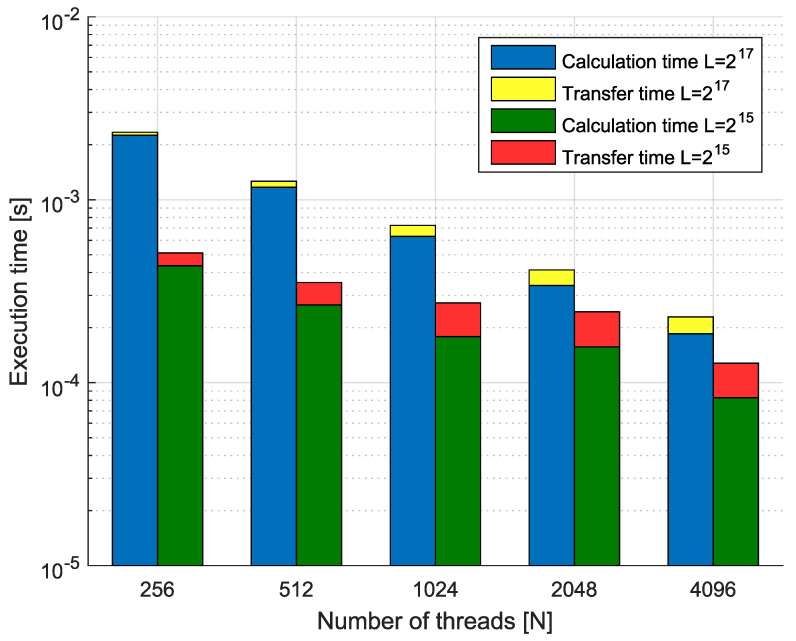
Calculation times for various numbers of threads.

**Example** **6.** *Consider the fractional-order state-space system presented in the introduction of this section. The system is calculated by use of parallelization Algorithm 3. Times of the one-step calculation process for implementation lengths $L=[2^{15},2^{17}]$ and various numbers of cores are presented in Figure 11. Moreover, Figure 11 presents data transfer times (red and yellow) and execution times (blue and green).*

*We can see that in the case of using GPU-based hardware and Algorithm 3, we obtain an effective tool for the distributed calculation of the fractional-order systems. For instance, increasing the number of processors 16 times (from 256 to 4096) for implementation length $L=2^{17}$ leads to speedup S=10.22. In contrast, for $L=2^{15}$ in the same case, we obtain S=4.00. Therefore, the parallelization process is much more effective for longer implementations of fractional-order systems. Additionally, we can see that the data transfer times are longer for high numbers of threads, but are still relatively short compared to the times of calculation.*

*Again, we compared the effectiveness of the proposed methodology with implicit GPU-support tools in the Matlab Parallel Computing Toolbox. Finally, we obtain execution times from $1.3\times 10^{-3}$ for $L=2^{15}$ to $3.0\times 10^{-3}$ for $L=2^{17}$. The times are higher in the case of the use of 256 workers for the hierarchical parallelization scheme introduced in the paper. Taking into account that Matlab is a high-level environment, where GPU-support is based on the same CUDA software as we use in our implementation, we can see that the Matlab parallelization algorithms are visibly less effective than for those considered in the paper.*

## 5. Conclusions

This paper has presented new parallelization algorithms for calculation of both the GL fractional-order derivative/difference and the fractional-order state-space system. For the fractional-order system, we introduce two different parallelization methods. The first method is dedicated to use with classical hardware and relatively low numbers of cores, and the second one is designed for Massively Parallel Processors. In simulation examples, we use computers with (a) an Intel Xeon E5−2650 v3 CPU (Intel Corporation, Santa Clara, CA, USA) and (b) Tesla K80 (Nvidia Corporation, Santa Clara, CA, USA) accelerators with a dual-GPU. Simulation examples confirm that the introduced methods can be effectively used in the accurate approximation of the fractional-order systems, in particular for high calculation lengths. The direction of our future research will be focused on numerical methods for solving fractional-order systems based on different fractional-order derivatives/differences, as well as their parallel implementation algorithms.
